# Sigma 1 Receptor Co-Localizes with NRF2 in Retinal Photoreceptor Cells

**DOI:** 10.3390/antiox10060981

**Published:** 2021-06-19

**Authors:** Shannon R. Barwick, Mevish S. Siddiq, Jing Wang, Haiyan Xiao, Brendan Marshall, Elizabeth Perry, Sylvia B. Smith

**Affiliations:** 1 Department of Cellular Biology and Anatomy, Medical College of Georgia at Augusta University, Augusta, GA 30912, USA; SBARWICK@augusta.edu (S.R.B.); mevishssiddiq@gmail.com (M.S.S.); JWANG1@augusta.edu (J.W.); HXIAO@augusta.edu (H.X.); BMARSHALL@augusta.edu (B.M.); LPERRY@augusta.edu (E.P.); 2 James and Jean Culver Vision Discovery Institute, Augusta University, Augusta, GA 30912, USA; 3 Department of Ophthalmology, Medical College of Georgia at Augusta University, Augusta, GA 30912, USA

**Keywords:** sigma receptor, NRF2, retinal degeneration, oxidative stress, electron microscopy (EM) immunodetection, retinal neuroprotection, pentazocine, mouse, photoreceptor cells, cone cells

## Abstract

Sigma 1 receptor (Sig1R), a modulator of cell survival, has emerged as a novel target for retinal degenerative disease. Studies have shown that activation of Sig1R, using the high affinity ligand (+)-pentazocine ((+)-PTZ), improves cone function in a severe retinopathy model. The rescue is accompanied by normalization of levels of NRF2, a key transcription factor that regulates the antioxidant response. The interaction of Sig1R with a number of proteins has been investigated; whether it interacts with NRF2, however, is not known. We used co-immunoprecipitation (co-IP), proximity ligation assay (PLA), and electron microscopy (EM) immunodetection methods to investigate this question in the 661W cone photoreceptor cell line. For co-IP experiments, immune complexes were precipitated by protein A/G agarose beads and immunodetected using anti-NRF2 antibody. For PLA, cells were incubated with anti-Sig1R polyclonal and anti-NRF2 monoclonal antibodies, then subsequently with (−)-mouse and (+)-rabbit PLA probes. For EM analysis, immuno-EM gold labeling was performed using nanogold-enhanced labeling with anti-NRF2 and anti-Sig1R antibodies, and data were confirmed using colloidal gold labeling. The co-IP experiment suggested that NRF2 was bound in a complex with Sig1R. The PLA assays detected abundant orange fluorescence in cones, indicating that Sig1R and NRF2 were within 40 nm of each other. EM immunodetection confirmed co-localization of Sig1R with NRF2 in cells and in mouse retinal tissue. This study is the first to report co-localization of Sig1R-NRF2 and supports earlier studies implicating modulation of NRF2 as a mechanism by which Sig1R mediates retinal neuroprotection.

## 1. Introduction

Mutations in >200 genes have been identified that lead to human blindness [1,2,3]. In treating these diseases, it may not be feasible to correct each genetic defect; however, amplifying cytoprotective pathways in retinal cells holds promise for individuals afflicted with severe retinopathy. A number of studies implicate oxidative stress in the pathoetiology of retinal degenerative disease [4,5,6,7,8,9]; thus, modulating oxidative stress may prove beneficial as a therapeutic target strategy. Our laboratory has been investigating a unique protein, sigma 1 receptor (Sig1R), as a promising target for severe retinopathy. Sig1R, a ubiquitously expressed 25.3 kDa transmembrane receptor protein, plays a role in attenuating cellular stress by yet uncertain mechanisms [10,11]. The receptor is an evolutionary isolate with no similarity to other human proteins. Rigorous analyses of its crystal structure in humans revealed a trimeric architecture with a single transmembrane domain in each protomer [12]. When originally discovered, Sig1R was considered an opiate receptor, but this was disproven. Sig1R has gained attention in the field of neuroscience and is implicated in human neurodegenerative diseases, including Alzheimer’s disease, Parkinson’s disease, and amyotrophic lateral sclerosis [13]. Activating Sig1R has proven beneficial in models of these diseases. As reviewed recently, a number of mechanisms have been postulated to account for neuroprotection afforded by Sig1R activation, including regulation of inositol 1, 4, 5-triphosphate (IP3) receptors to ensure proper Ca^2+^ signaling, modulation of ER stress via the master ER stress regulatory protein BiP/GRP78, and regulation Bcl-2, an antiapoptotic protein, by induction of NFκB [11].

Retinal degeneration, a major cause of untreatable blindness, is a subset of neurodegenerative diseases. As with other neurodegenerative diseases, activation of Sig1R is beneficial in models of retinal degeneration [14]. One of the consequences of Sig1R activation, which is observed in multiple tissue types and is particularly relevant to retinal degenerations, is marked attenuation of levels of reactive oxygen species (ROS) [15,16,17]. Several studies suggest that a novel mechanism by which Sig1R attenuates ROS is modulation of nuclear factor erythroid 2-related factor 2 (NRF2) [18,19,20]. NRF2 is a basic leucine zipper transcription factor responsible for regulating transcription of more than 500 antioxidant and cytoprotective genes [21]. In the absence of overt stress, NRF2 is sequestered in the cytosol by its repressor protein Kelch ECH-associating protein 1 (KEAP1); excess NRF2 is degraded by the proteasome. Increased cellular stress causes a conformational change in the NRF2–KEAP1 complex, releasing NRF2 and allowing it to translocate to the nucleus, where it heterodimerizes with small Maf proteins and subsequently binds to ‘antioxidant response elements’ (ARE), cis-acting regulatory enhancers found in the 5′ flanking region of many phase II detoxification enzymes and antioxidant proteins [22]. Evidence of a relationship between Sig1R and NRF2 has been shown in several studies. For example, activation of Sig1R with (+)-pentazocine ((+)-PTZ), a high-affinity, high-specificity Sig1R ligand, increases binding of NRF2 to AREs in AREc32 cells stably expressing ARE-Luc [18]. A dose-dependent increase in NRF2 protein levels (as well as Sig1R protein) was detected when Sig1R was activated using increasing concentrations of (+)-PTZ in the 661 W retinal photoreceptor cell line [18]. Similar findings were observed in experiments performed in astrocytes cultured from rats using SA-4503, a selective Sig1R agonist [20]. In that study, astrocytes treated with lipopolysaccharide (LPS), a known inducer of the inflammatory response and oxidative stress, showed decreased NRF2 levels, whereas SA-4503 treatment significantly reduced LPS-induced oxidative stress and upregulated NRF2 signaling. Studies performed in primary retinal Müller glial cells harvested from *Sig1R^-/-^* mice showed a marked increase in endogenous ROS levels and decreased NRF2 expression at the gene and protein levels [17]. Ruoho’s lab reported elevated ROS levels in livers harvested from *Sig1R^-/-^* mice and demonstrated that Sig1R ligands activate AREs in COS cells [16]. The notion of a relationship between Sig1R and NRF2 was further strengthened by the observation that silencing *Sig1R* decreased NRF2 protein levels (as well as Sig1R) [18]. Taken together, these findings provide evidence that activation of Sig1R modulates NRF2 activity or expression. Despite these studies, it is not clear whether Sig1R resides within cells in close proximity to NRF2. Sig1R is located in the cytoplasm (at the ER–mitochondrial membrane), as well as the nuclear membrane [15,23,24]. It interacts with many proteins (~50 have been experimentally determined). Given the extensive evidence that activation of Sig1R modulates NRF2, which also has a cytoplasmic and nuclear location, we investigated in the present study whether the two proteins co-localize.

## 2. Materials and Methods

### 2.1. Cell Culture

The 661 W mouse cone photoreceptor cell line (obtained from Dr. M. Al-Ubaidi, University of Houston), expresses blue and green cone pigments, transducin, and cone arrestin, which are proteins expressed in cone photoreceptors [25]. The 661 W cells were cultured in Dulbecco’s modified Eagle’s medium (DMEM, Thermo Fisher Scientific, Waltham, MA, USA, Cat. No. 10-013-CV) supplemented with 1% FBS, 100 U/mL penicillin, and 100 μg/mL streptomycin.

### 2.2. Protein Complex Immunoprecipitation (Co-IP) of Sig1R and NRF2

The 661 W cells were lysed in immunoprecipitation lysis buffer (Pierce, Thermo Fisher Scientific, Cat. No. 87787) containing Halt Protease/Phosphatase Inhibitor Cocktail (Cat. No. 1861581) according to the manufacturer’s instructions. Cellular lysate supernatant was obtained via centrifugation (14,000× *g*, 10 min, 4 °C) until reaching pellet cellular debris. Protein concentration was determined using the BCA kit (Pierce, Cat. No. 23227). For immunoprecipitation of Sig1R, freshly prepared samples containing 500 µg of total protein were pre-cleaned via incubation with protein A/G agarose beads (Santa Cruz, CA, USA) for 30–60 min at 4 °C. The supernatant was incubated with anti-Sig1R using a rabbit polyclonal antibody generated in our laboratory [26] at a dilution of 1:25 at 4 °C overnight. The specificity of the anti-Sig1R antibody has been confirmed [27]. Immune complexes were precipitated by protein A/G agarose beads at 4 °C for 2 h. The immunoprecipitates were washed 5× with lysis buffer on ice and re-suspended with Laemmli sample buffer (BioRad, Hercules, CA, USA, Cat. No. 161-0737). The samples were separated by SDS-PAGE (4–15% gradient gel), transblotted onto nitrocellulose membranes, immunodetected using anti-NRF2 antibody (R&D, MAB3925), and verified using a mouse anti-Sig1R antibody (Santa Cruz, Santa Cruz, CA, USA, sc-137075). In addition, the whole cell lysates were subjected to immunoblotting to detect Sig1R and NRF2 as described [18]. GAPDH (EMD Millipore, Burlington, MA, USA, MAB374) was used as the protein loading control. Data are representative of three independent experiments.

### 2.3. Proximity Ligation Assay (PLA)

The 661 W cells were seeded in eight-well chamber slides (Lab-Tek II CC Chamber Slide^TM^) and grown for 24 h. They were incubated in the presence or absence of (+)-PTZ (Sigma-Aldrich, St. Louis, MO, USA, Cat. No. 7361-76-4) (20 µM), prepared in 10% DMSO in 0.01 M PBS for 3 h or 24 h, fixed in 4% paraformaldehyde (Electron Microscopy Science (EMS), Hatfield, PA, UK) for 10 min, and washed with PBS. Cells were permeabilized for 10 min using 20% Triton-X100. The proximity of Sig1R to NRF2 was assessed using the Duolink^®^ PLA In Situ Orange Starter Kit (Mouse/Rabbit) (Sigma-Aldrich, St. Louis, MO, USA). Blocking solution provided with the kit was added and slides were incubated in a humidified chamber at 37 °C for 60 min. Slides were incubated with polyclonal rabbit Sig1R antibody (1:1000) and monoclonal mouse NRF2 antibody (1:500) overnight at 4 °C. Subsequently, slides were washed for twice for 5 min in 1× wash buffer A at room temperature, followed by incubation with Duolink PLA PLUS and MINUS probes diluted 1:5 in Duolink antibody diluent at 37 °C for 1 h. Then, 5× Duolink Ligation buffer was diluted in high-purity water at a 1:5 ratio and slides were washed. Slides were incubated in ligation buffer for 30 min at 37 °C. All subsequent steps were performed in the dark. The 5× amplification buffer was diluted 1:5 in high-purity water. Slides were washed as above. Polymerase was added to the diluted amplification buffer (1:80) and slides were incubated at 37 °C for 100 min. Slides were washed twice for 10 min with 1× wash buffer B, with one final wash for 1 min in 0.01× wash buffer B. In conjunction with these steps, we used the Duolink PLA control kit (PPI; Sigma-Aldrich) to ensure that our assay was working, and in separate studies we omitted the primary antibodies to evaluate artefactual fluorescence. Cover slips were mounted on slides using Duolink In Situ Mounting Medium with DAPI and sealed. Fluorescent images were captured using the Zeiss Axio Imager D2 microscope (Carl Zeiss, Göttingen, Germany) and a Zeiss Axiocam 305 camera equipped with ZenPro software. Data are representative of three independent experiments.

### 2.4. Electron Microscopic Immunogold Detection of Sig1R and NRF2

The 661 W cone cells were centrifuged (500× *g*, 10 min), fixed in 4% formaldehyde 0.2% glutaraldehyde in 0.1 M sodium cacodylate buffer at pH 7.4, dehydrated in graded ethanol through 95%, and embedded in LR White resin (EMS). Then, 70 nm sections were cut with a diamond knife on a Leica EM UC7 ultramicrotome (Leica Microsystems, Inc., Bannockburn, IL, USA) and collected on nickel grids. Sections were etched in 5% sodium metaperiodate (15 min) and washed 3 × 5 min with PBS, quenched in 1 M ammonium chloride in PBS, and washed for 3 × 5 min with PBS. Sections were blocked in Aurion blocking solution for goat gold conjugates (Electron Microscopy Sciences, Hatfield, PA, UK) for 2–4 h, then placed in diluted first primary antibody (antimouse NRF2) solution overnight at 4 °C. Sections were rinsed in PBS for 5 × 10 min and placed in antimouse Nanogold (1:1000) (Nanoprobes, Inc., Yaphank, NY, USA) in Aurion BSA-C buffer (Electron Microscopy Sciences) in PBS for 2 h at RT. Sections were washed for 5 × 10 min in PBS, for 2 × 10 min in de-ionized water (DI H_2_O), then silver-enhanced for 12 min in HQ Silver (Nanoprobes, Inc.) This enhancement resulted in large gold particles labeling the first primary antibody. Sections were washed in ice-cold H_2_O for 3 × 5 min to stop enhancement. Subsequently, sections were placed in diluted second primary antibody (antirabbit Sig1R), following the same steps as above for the first primary antibody and the secondary nanogold antibody until the second enhancement step. The sections were then enhanced with HQ Silver (Nanoprobes, Inc.) for 6 min, resulting in small gold particle labeling of the second primary antibody. The result was two different sizes of gold particles, with the larger representing NRF2 and the second smaller particle representing Sig1R. Sections were washed for 3 × 5 min with DI H_2_O and stained for 5 min with 2% aqueous uranyl acetate. Grids were allowed to dry and cells were observed in a JEM 1230 transmission electron microscope (JEOL USA, Inc., Peabody, MA, USA) at 110 kV, then imaged with an UltraScan 4000 CCD camera and First Light Digital Camera Controller (Gatan, Inc., Pleasanton, CA, USA). Twenty cells were evaluated for co-localization.

To confirm the EM immunodetection, we also performed colloidal gold labeling. While colloidal gold labeling has the disadvantage of poorer penetration [28], it allows verification because the sizes of the gold particles that label the antibody of interest are known precisely. It serves as a useful adjunct to the more commonly used nanogold labeling described above. Sections were incubated in blocking buffer (5% BSA, 3% normal serum, 0.05% Tween-20 in Tris-buffered saline, pH 7.4) at RT in a humidified chamber for 2 h, followed by incubation with primary antibody diluted in blocking buffer overnight at 4 °C. Grids were washed and incubated for 2 h at RT with colloidal-gold-labeled secondary antibody (for SIG1R, antirabbit 18 nm colloidal gold; for NRF2, antimouse 6 nm colloidal gold), then washed and stained with 2% alcohol uranyl acetate and 0.08% alkaline bismuth subnitrate. Cells were analyzed using the JEM 1230 scope as described above. Fifteen cells were evaluated for co-localization.

### 2.5. EM Evaluation of Sig1R and NRF2 in Mouse Retinal Tissue

C57BL/6J mice (aged 6–8 weeks) were obtained from The Jackson Laboratories (Bar Harbor, ME, USA). They were euthanized and eyes were harvested for ultrastructural localization of Sig1R and NRF2. Eyes were fixed (4% paraformaldehyde, 2% glutaraldehyde in 0.1 M cacodylate buffer, pH 7.4), processed through graded ethanol and LR White resin, and collected on nickel grids. Tissues were processed for nanogold labeling, additional tissues were processed for colloidal gold labeling. They were visualized by transmission EM as described for cells. Oversight of the animals used in this study followed our IACUC-approved protocol and was consistent with the ARVO statement for Use of Animals in Ophthalmic and Vision Research. Retinas from three (3) mice were used in the analysis.

## 3. Results

### 3.1. Co-Immunoprecipitation Analysis of Sig1R and NRF2

**Figure 1:**
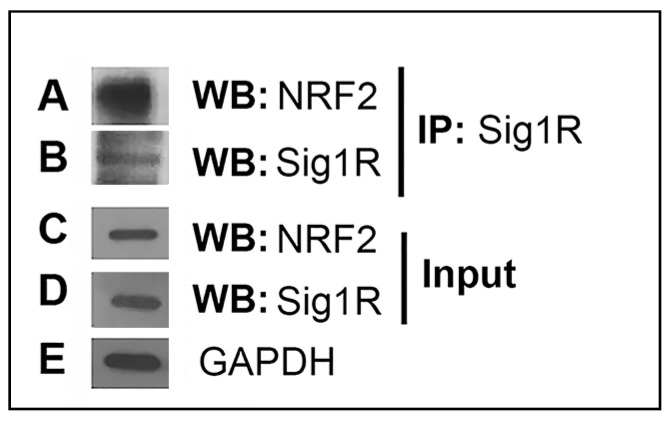
Co-immunoprecipitation of Sig1R and Nrf2 in 661 W cells. Lysates of 661 W cells were incubated with anti-Sig1R antibody and immune complexes were precipitated by protein A/G beads. The Sig1R complex was subjected to immunoblotting to detect (**A**) NRF2 and (**B**) Sig1R in the complex. The whole cell lysate was used for immunoblotting to detect (**C**) NRF2 and (**D**) Sig1R. (**E**) GAPDH served as an internal reference for sample loading.

A number of studies have suggested a relationship between Sig1R and NRF2 [18,19,20,29,30]. Here, we investigated whether such a relationship exists in retina. We used the 661 W retinal cone photoreceptor cell line to determine whether NRF2 is present in a complex with Sig1R using co-IP methods. The 661 W cells demonstrate robust expression of both proteins [18]. Co-IP is a commonly used method to identify physiologically relevant protein–protein interactions by using target-protein-specific antibodies to indirectly capture proteins to which it is bound. Sig1R protein was immunoprecipitated from the supernatant using anti-Sig1R antibody followed by agarose beads. Western blot analysis detected NRF2 in the Sig1R–bead complex from the IP procedure (Figure 1A), as well as Sig1R itself (Figure 1B). The analysis suggested that the two proteins were in a complex with each other. Western blot analysis of the whole cell lysate detected both NRF2 and Sig1R in the cells (Figure 1C,D). The loading control GAPDH was detected in abundance (Figure 1E).

### 3.2. Proximity Ligation Assay for Sig1R and NRF2

**Figure 2:**
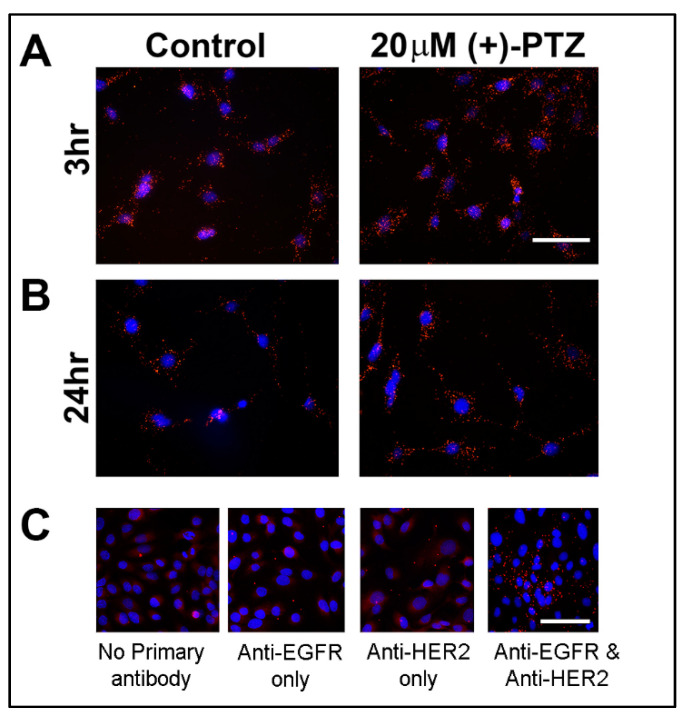
Proximity ligation assay (PLA) for Sig1R and Nrf2 in 661 W cells. The 661 W cells were incubated in the presence or absence of (+)-PTZ (20µM) for (**A**) 3 h or (**B**) 24 h. Cells were subjected to the PLA assay using rabbit anti-Sig1R and mouse anti-NRF2. Fluorescent images were captured and orange-red puncta represent co-localization of Sig1R and NRF2. To confirm the validity of the assay, we used the Duolink PLA Control Kit, which provides SK-OV-3 human ovarian cancer cells that were treated with EGF to detect two proteins known to co-localize following EGF treatment (EGFR and HER2). (**C**) When no antibody was present or when only one antibody was present (anti-EGFR only, anti-HER2 only), no orange-red fluorescent puncta were detected; however, when both antibodies (anti-EGFR and anti-HER2) were present in the assay, abundant orange puncta were visible. DAPI (4′, 6-diamidino-2-phenylindole), a fluorescent stain, was used to detect cell nuclei. Calibration bar = 30 µm.

To further investigate the relationship between Sig1R and NRF2, we exploited PLA, a powerful tool that permits in situ detection of proteins that are proximally close to each other. The assay requires antibodies raised in different species to detect unique protein targets. In our studies, we used an antibody against Sig1R raised in rabbit and an antibody against NRF2 raised in mouse. A pair of oligonucleotide-labeled secondary antibodies (the PLA probes, which are provided in the kit) binds to the primary antibodies. The basis of the assay is that the hybridizing connector oligos join the PLA probes only when the proteins are in close proximity to each other (<40 nm). This is observed as punctate orange-red fluorescence. We used the Duolink PLA control kit (PPI) and established the validity of the assay. The 661 W cells cultured in the presence or absence of (+)-PTZ (20 μM) for 3 or 24 h were processed for PLA using antibodies against Sig1R and NRF2; fluorescent images were captured. We observed orange-red fluorescent puncta in cells treated for 3 h (Figure 2A) and 24 h with (+)-PTZ (Figure 2B). The fluorescence was most obvious in the cytoplasm. It is noteworthy that non-treated (control) cells also showed orange-red fluorescence; thus, regardless of whether cells had (or had not) been exposed to (+)-PTZ for either 3 or 24 h, there was an abundance of fluorescence consistent with Sig1R residing in 661 W cells in close proximity to NRF2. These data offered additional support to the suggestion that Sig1R and NRF2 co-localize. Separate experiments omitted the primary antibodies and no orange fluorescent puncta were detected. We validated the PLA assay using the Duolink PLA protein–protein interaction system. This system emits orange fluorescent puncta characteristic of two proteins interacting (EGFR and HER2) when the cells are treated with EGF (Figure 2C). PLA is a useful tool for interrogating the proximity of one protein to another; however, it is not without limitations. Due to saturation effects, PLA is recommended as a semi-quantitative tool for assessing protein associations [31]; therefore, we performed EM immunolocalization analyses to confirm our findings.

### 3.3. Electron Microscopic Immunodetection of Sig1R and NRF2

**Figure 3:**
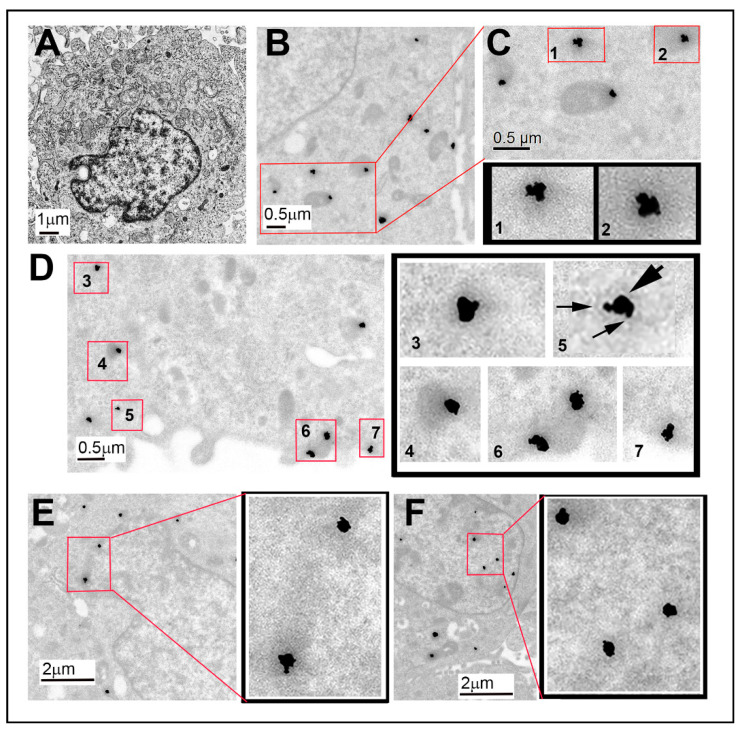
EM immunodetection of Sig1R and NRF2 in cultured 661 W photoreceptor cells. The 661 W cells cultured as described in the text were processed for post-embedding EM immunolocalization to detect Sig1R and NRF2 using nanogold-labeled secondary antibodies. (**A**) Low-magnification image of a typical cell shown adjacent to higher magnification images of the cytoplasm (**B**–**D**) and the nucleus (**E**,**F**). (**B**) An area encased in red, which is magnified in (**C**); wo clusters are shown at even higher magnification (panels **C1**,**C2**). The clusters are gold particles reflecting the two proteins of interest (NRF2 (large, electron-dense gold nanoparticles) and Sig1R (smaller, electron-dense gold nanoparticles)). (**D**) Five clusters (numbered **D3**, **D4**, **D5**, **D6**, **D7**) are shown at higher magnification in the adjacent panel. (**E**) An area of the nuclear membrane; two clusters are outlined in red, which are enlarged in the adjacent panel (outlined in black) (**F**) Clusters within the nucleus outlined in red and enlarged in the adjacent panel. The data reflect co-localization of NRF2 and Sig1R.

Post-embedding EM immunolocalization methods were used to evaluate whether Sig1R and NRF2 were within close proximity to each other in 661 W cells. Since the PLA study showed no difference in co-localization in the presence or absence of (+)-PTZ, most of the EM studies were performed without (+)-PTZ treatment. A representative electron micrograph from cells prepared in Embed 812 and stained with osmium tetroxide is shown as a reference to allow visualization of the cell nucleus and organelles within the cytoplasm (Figure 3A). The remaining panels in Figure 3 show immunodetection of the two proteins in cells processed in LR White. There were instances of co-localization in the cytoplasm (Figure 3B–D), as well as in the nucleus and nuclear membrane (Figure 3E,F). Regarding the co-localization in the cytoplasm, low-magnification images revealed clusters of gold particles (Figure 3B). The red inset in Figure 3B is enlarged in Figure 3C and enlarged again in panels 1 and 2 within Figure 3C. At this very high resolution, electron-dense particles of two sizes reflecting the two proteins can be seen (larger particle representing NRF2 and smaller particle representing Sig1R). Figure 3D shows several other areas (within red-bordered boxes) within the cytoplasm with electron-dense clusters, which are enlarged in the adjacent panel and labeled (panels 3–7). In Figure 3D (panel 5), arrows point to the smaller and larger gold particles clustered together, reflecting co-localization of the two proteins. Co-localization was also observed in the nucleus and the nuclear membrane. Figure 3E and Figure 3F show low-magnification images and much higher magnification images, respectively, reflecting the abundance of small–large particles detected in clusters. We confirmed the findings using colloidal gold immunolabeling (Supplementary Figure S1).

**Figure 4:**
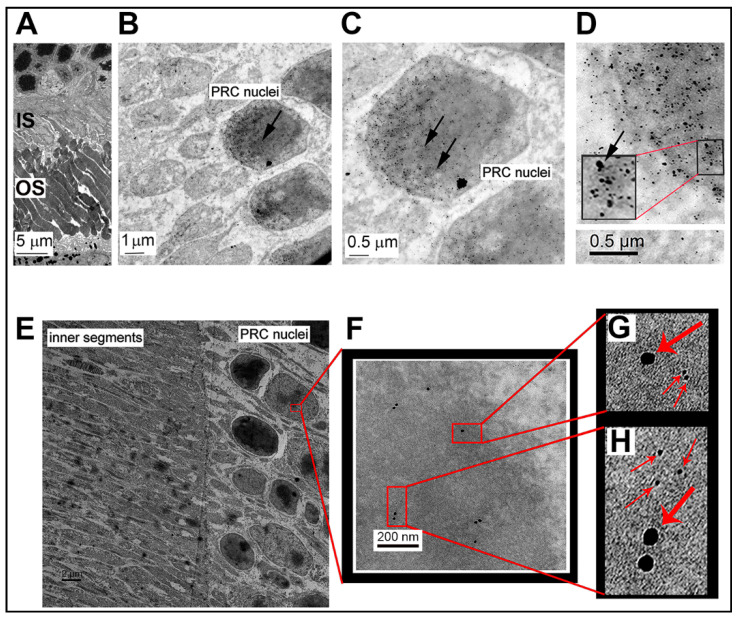
EM immunodetection of Sig1R and NRF2 in photoreceptor cells of WT mouse retinas. WT mouse retinas were processed for post-embedding EM immunolocalization to detect Sig1R and NRF2 using nanogold- (**A**–**D**) or colloidal-gold-labeled (**E**–**H**) secondary antibodies. (**A**) Low-magnification image of the outer portion of the retina, specifically the inner segments and adjacent photoreceptor cell nuclei. Increasingly higher magnifications are shown in (**B**–**D**). The nanogold labeling of larger and smaller particles reflects NRF2 and Sig1R, respectively, as explained in the methods section. (**D**) The clusters of large and small particles demonstrate an abundance of co-localization between Sig1R and NRF2 in the photoreceptor cell nuclei. Data from colloidal gold labeling confirm the nanogold labeling. (**E**) Low-magnification image of photoreceptor cell nuclei and adjacent inner segments. (**F**) Higher magnification of a single photoreceptor cell nucleus with colloidal gold particles evident. (**G**,**H**)Higher magnification insets from (F) show the close proximity (<50 nm) of the two differently sized gold particles, confirming the close proximity of Sig1R (18 nm) and NRF2 (6 nm) in the photoreceptor cell. Abbreviations: IS = inner segments; OS = outer segments; PRC = photoreceptor cell.

The data described in Figure 3 were generated in a cone photoreceptor cell line under in vitro conditions. To investigate whether Sig1R co-localized with NRF2 in intact retina, we harvested eyes from WT adult mice and processed them for embedding in LR White. Retinas were mounted on grids and post-embedding immunodetection was performed as described for the studies in cells. Representative electron micrographs show the organization of the photoreceptor cell nuclei and the adjacent inner and outer segments in Figure 4A. Nanogold immunodetection of the two proteins in photoreceptor cell nuclei of the intact retina is shown at low magnification in Figure 4B and at higher magnification in Figure 4C,D. There are dense clusters of gold particles visible in photoreceptor cell nuclei in Figure 4C,D. Arrow heads point to these particles in Figure 4C, which are clearly of small and large diameters consistent with labeling of the two proteins. At very high resolution in Figure 4D, the electron-dense particles reflect the two proteins (with larger particles representing NRF2 and smaller particles representing Sig1R). The inset in Figure 4D illustrates this clearly. We confirmed that Sig1R and NRF2 were in close proximity using the colloidal gold immunolabeling method, which offers precise detection of a protein because the gold particle size is exact, despite the fact that it affords poor penetration of gold particles into tissue. Representative electron micrographs show the organization of the photoreceptor cell nuclei and the adjacent inner segments in Figure 4E. With increasing magnification (Figure 4F), it was possible to visualize Sig1R (18 nm) and NRF2 (6 nm) gold nanoparticles that were within 50 nm of each other. This close proximity can be seen more clearly at even higher magnification in Figure 4G,H; thus, in the retina, there was clear evidence of co-localization of Sig1R and NRF2 in photoreceptor cells.

## 4. Discussion

This work was undertaken to investigate whether Sig1R and NRF2 co-localized in retinal photoreceptor cells. Earlier, we observed dramatic rescue of the cone photoreceptor function in the *Pde6b^rd10^/J* (rd10) mouse model of retinitis pigmentosa when we treated the animals with the potent Sig1R ligand (+)-PTZ [30]. Among the findings from that study was clear evidence that (+)-PTZ treatment attenuated oxidative stress. Earlier studies have clearly shown that NRF2 levels are modulated by activation of Sig1R [18]; however, it had not been determined whether these two proteins are actually in close proximity to each other.

We performed experiments in the 661 W cell line using co-immunoprecipitation methods, proximity ligation assays, and EM immunolocalization methods. The data supported the notion that the two proteins co-localize. First, the co-immunoprecipitation experiment suggested that NRF2 was bound in a complex with Sig1R. The PLA study suggested co-localization of the two proteins within ~40 nm (the level of resolution of the kit used for this experiments), while the EM study revealed numerous instances of clusters representing the two proteins. Retinal tissue harvested from WT mice supported the close proximity of Sig1R and NRF2 in photoreceptor cell nuclei.

Sig1R is an enigmatic protein whose biological function is a matter of debate. It is expressed ubiquitously and is located in several cellular organelles, including the ER, mitochondrial-associated membrane, and nucleus. NRF2 is a key molecule involved in the response to oxidative stress. It is present typically in the cytoplasm (tethered to KEAP1) but translocates to the nucleus to activate AREs of numerous antioxidant genes. Our interest in whether these two proteins co-localize stems from experiments showing that when Sig1R is activated in cells, there is an increase in NRF2–ARE binding activity, *Nrf2* gene expression, and NRF2 protein levels [18], whereas silencing *Sig1R* is accompanied by decreased NRF2. In vivo studies conducted to investigate whether activation of Sig1R in the absence of NRF2 rescues cones in a mutant model of retinal degeneration revealed that Sig1R-mediated cone rescue requires NRF2 [18].

Sig1R is considered a modulator of cellular signaling pathways, particularly G-coupled receptor and ion channel signaling. In an effort to elucidate the function of Sig1R, many experiments have been performed to assess whether Sig1R interacts with specific proteins. While the individual studies are far too numerous to cite, two review papers provided an excellent overview of the literature related to proteins that interact with Sig1R [10,11]. Included among those are proteins that reside at the plasma membrane (e.g., acid-sensing ion channels, dopamine receptors, muscarinic acetylcholine receptors, mu opioid receptors, NMDA receptors, cannabinoid receptor 1, tropomyosin receptor kinase B (TrkB), platelet-derived growth factor receptor, and voltage-gated potassium and sodium channels). In the cytoplasm, proteins that are thought to interact with Sig1R include ELMOD (cell engulfment and motility domain) proteins and Rac-GTPase. At the ER–mitochondrion interface and mitochondria are BiP/GRP78, IP3R3, ankyrin, voltage-dependent anion channels, IRE1, and insulin-induced gene. Regarding possible interaction partners of Sig1R in the nucleus, emerin has been shown to co-localize with Sig1R.

To date, Sig1R has been reported to bind to nearly 50 proteins of varying sequences and structures. The most common methods for interrogating the binding have been co-IP and PLA. We submit here that based on co-IP, PLA, and EM-immunolocalization methods, another protein that co-localizes with Sig1R is NRF2. It is clear from earlier studies that modulating Sig1R impacts the function of NRF2 [17,18,19,20,29]. The reason for these findings may be due in part to the close proximity of the proteins.

In terms of the limitations of this study, we acknowledge that antibodies are important for the work presented here. In our laboratory, we generated an antibody against Sig1R several years ago and validated it using *Sig1R^-/-^* mouse tissues [26,27]. We have experimented with NRF2 antibodies from many sources and tested each of them in retinas of WT vs. *Nrf2^-/-^* mice [17,18]. We successfully detected NRF2 using antibodies purchased from Abcam Corp. and from R&D Systems. Unfortunately, Abcam is no longer producing their antibody. In a previous study, the researchers actually attempted to perform co-IP of Sig1R and NRF2 and indicated their difficulties with the NRF2 antibody from Cell Signaling Technology (Danver, MA, USA) [29]. The authors had to use a protease inhibitor and Sig1R overexpression to perform their work in primary astrocytes. We appreciate the challenges they experienced, but in our hands the 661 W cells and the mammalian retina both express NRF2 at detectable levels, fortunately not requiring either of these efforts.

Regarding a possible interaction with NRF2, the data we provide here suggest that it co-localizes with Sig1R. While activation of Sig1R clearly alters activity and expression levels of NRF2, it remains to be tested experimentally whether there is a direct interaction between the two proteins. Nevertheless, the data emanating from several labs suggest an important relationship between Sig1R and NRF2 that may, at least in part, explain its neuroprotective properties.

## 5. Conclusions

Sig1R is a promising therapeutic target for retinal disease. While earlier studies suggested that a mechanism by which Sig1R mediates retinal disease involves the key antioxidant protein NRF2, it had not been determined whether these two proteins interact. The present study addressed this using co-IP, PLA, and two EM immunodetection methods. The data obtained in a retinal photoreceptor cell line and in retinal tissue suggested that the two proteins co-localize, supporting the notion that modulation of NRF2 is involved in Sig1R-mediated retinal neuroprotection.

## Data Availability

All methods and protocols used in this study will be made available to researchers upon request.

## Supplementary Materials

The following are available online at https://www.mdpi.com/article/10.3390/antiox10060981/s1: Figure S1: Colloidal gold immunodetection of Sig1R and NRF2 in cells cultured with (+)-PTZ.
