# Supplementary figures and images for: Sigma 1 Receptor Co-Localizes with NRF2 in Retinal Photoreceptor Cells
Source: Antioxidants (Basel). 2021 Jun 19;10(6):981. doi: 10.3390/antiox10060981 (PMC8234060; doi:10.3390/antiox10060981)

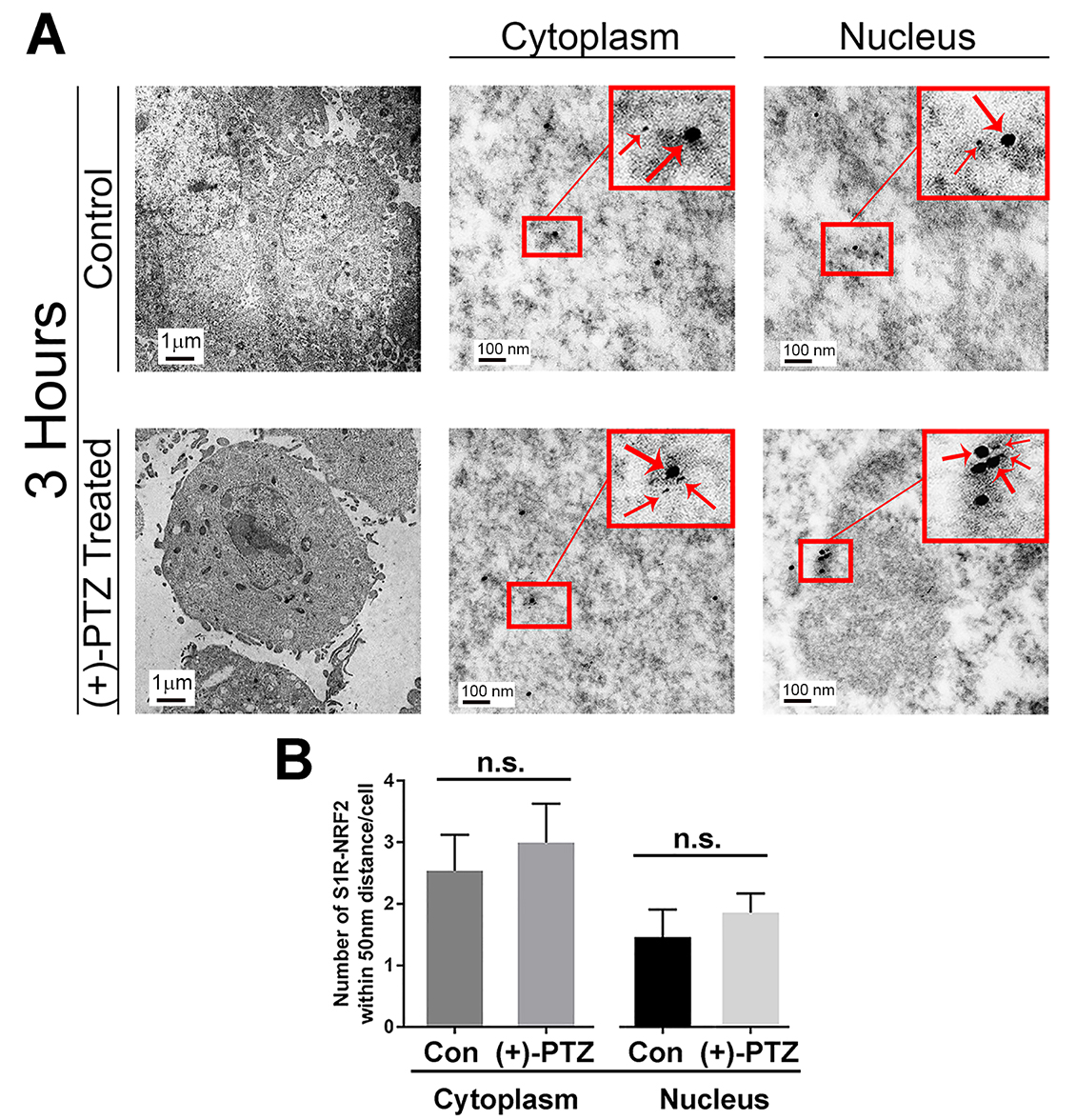

Supplement: Supplementary file 1 [file antioxidants-10-00981-s001.zip › antioxidants-1256130-supplementary.jpg]
